# Scientific clues on global food (in)security and climate change relationship as drivers of health: Dilek Aslan

**DOI:** 10.1093/eurpub/ckac130.073

**Published:** 2022-10-25

**Authors:** D Aslan

**Affiliations:** Department of Public Health, Hacettepe University Faculty of Medicine, Sihhiye-Ankara, Turkey

## Abstract

**Issue:**

Food insecurity is in close relationship with the determinants of health. Global crisis including climate change (CC), natural disasters, poverty can deepen the burden, and all are linked with the economic, social, commercial, structural determinants of health. Defining such connections may help in proposing practical solutions.

**Description of the problem:**

COVID-19 pandemic made the food security (FS) problem more visible. Food security considers basically the affordability, availability, and the quality of food. Food insecurity (FiS), violation of the right to healthy food, influences disease patterns and causes communicable and non-communicable diseases (NCDs). Globally, 71% of deaths are attributed to NCDs. Analyzing the relationship between FiS and other determinants of health like CC may be helpful for sustainable solutions in such a world where we are talking on “our planet, our health” motto. Example given in this study is the relationship between the country values/rankings of the “Global Food Security Index (GFSI)” and the “Climate Change Performance Index (CCPI)”. GFSI defines the FS situation and CCPI defines countries’ response to CC.

**Results:**

Countries’ CCPI and GFSI values do not show a linear relationship. For example, Norway, as a country at the top of the Human Development Index (HDI) ranking list has both high CCPI and GFSI values. On the other hand, although USA and Canada have low CCPI, both have good GFSI values. Sub dimensions of the indicators may also vary across countries. Crisis like COVID-19, conflicts, poverty emphasize the need on improving the indicators in a transdisciplinary approach.

**Lessons:**

Investigating indicators taking the determinants of health into account is helpful. However, different characteristics of the countries make it difficult to propose a standard approach to overcome the problems. Developing “new” indicators with transdisciplinary work might be useful in this sense.

**Key messages:**

- Food (in)security and climate change have “complex” interactions.
- Transdisciplinary approach may facilitate proposing realistic solutions.

